# Active delivery of the anterior arm and incidence of second-degree perineal tears: a clinical practice evaluation

**DOI:** 10.1186/s12884-017-1322-8

**Published:** 2017-05-12

**Authors:** Nicolas Mottet, Marine Bonneaud, Astrid Eckman-Lacroix, Rajeev Ramanah, Didier Riethmuller

**Affiliations:** Department of Obstetrics and Gynecology- Besancon University Medical Center, CHRU Jean Minjoz, Alexander Fleming Boulevard, 25000 Besancon, France

**Keywords:** Levator ani muscle injury, Perineal laceration, Vaginal delivery

## Abstract

**Background:**

Evaluate the feasibility of active delivery of the anterior arm during spontaneous delivery. This maneuver could decrease incidence of second-degree perineal tears because it reduces fetal biacromial diameter.

**Methods:**

An observational comparative prospective study was conducted at our teaching maternity from July 2012 to March 2013. The study included 199 nulliparous women ≥18 years, who met the following criteria: singleton pregnancy, vaginal delivery with occiput anterior presentation, on epidural analgesia, from 37 weeks of gestation onward. The distribution of rate and type of perineal tears were compared between two groups: a non-exposed group and a group exposed to the maneuver.

**Results:**

A total of 101 patients were exposed to Couder’s maneuver (CM) and 98 patients were not exposed. In the intervention group, 3 failures of the maneuver were reported. The maneuver was considered easy in 80% of cases, moderately easy in 12% and difficult in 8% of cases. There was a significant difference (*p* = 0.03) in the distribution of perineal tears between the two groups. There was a significant reduction (*p* < 0.001) in the number of second-degree perineal tears in the patients exposed to CM. There was no significant difference in the rate of anterior perineal trauma between the exposed and non-exposed arms.

**Conclusions:**

CM in primiparous women at term is feasible with a low failure rate and influences the distribution of perineal tears by lowering second-degree perineal tears in a highly significant manner (p <0.01).

**Electronic supplementary material:**

The online version of this article (doi:10.1186/s12884-017-1322-8) contains supplementary material, which is available to authorized users.

## Background

The last few years, much attention has been focused on obstetric anal sphincter injuries prevention and the indications for episiotomy. However, levator-ani muscle injuries form an important component of pelvis floor trauma and occur in 13 to 36% of women who deliver vaginally [1–5]. These lesions increase the risk of cystocele and uterine prolapse according to a literature review by Schwertner et al. [6].

Promoting obstetric maneuvers that improve visual and manual perineal management appears to be necessary to prevent perineal trauma during fetal head and shoulders delivery. Initially in Ritgen’s maneuver, the fetal chin is pulled interiorly to keep flexion of the fetal head and control speed of delivery between contractions. But actually, some practices of this maneuver deviate from its initial description. Authors describe a modified Ritgen’s maneuver during contractions [7] and others use this maneuver by hooking the chin increasing the diameter oh the head on the perineum [8] . What actually does show the most significant reduction in severe perineal tears is the Finnish method and a complete teaching package [9–12]. But no systematic reviews have been published comparing different perineal support during the second stage of labour for reducing perineal trauma [13].

Delivery of the infant’s shoulders is usually assisted by downward traction first to free the anterior shoulder. Then, the posterior shoulder is delivered with a risk of perineal tears because of tension on the perineum already weakened by fetal head delivery. Management of shoulders during delivery is a research area to improve perineal care. Active delivery of the anterior arm with Couder maneuver (CM) can be benefit because it has the advantage of reducing fetal biacromial diameter. This maneuver is specially described to manage shoulder dystocia especially when the posterior shoulder is in the pelvis and the anterior shoulder is wedged against the pubis [14] . In our center, CM is usually performed during normal deliveries, but its impact on perineal tears has never been described.

The objective of this study was to evaluate the feasibility of CM and its impact on second-degree perineal tears. There has so far been no report in the literature on the usefulness of this maneuver.

## Methods

### Study Design

We carried out a prospective comparative non-randomized study in our teaching high-risk maternity from July 2012 to March 2013. *The study respected ethical rules set by our local ethical committee* (Institutional Review Board of Besancon University Medical Center “*Comité Protection des Personnes CPP EST II* EST-II”). The study took place at the department of Obstetric at Besancon University Medical Center, with an obstetric unit with 2,800 yearly deliveries. The study adhered to STROBE guidelines. Women likely to be included in the study were informed of the study and modalities of CM during the last medical visit with a recall at admission in the delivery room. An information sheet explaining the aim and usefulness of the study was given to them. All volunteers gave written informed consent.

A group of consecutive women exposed to CM according to our clinical practice was compared to a control group with spontaneous delivery of the shoulder. Women were enrolled if they agreed to participate and were divided into two groups based on their choice to receive or not the maneuver. A control group of consecutive women with a spontaneous delivery was collected. The two groups were compared for age, weight gain, BMI, maternal comorbidities and new borns characteristics (Weight, bi-acromial diameter, Apgar score).

### Participants

The population of this study was women with a spontaneous vaginal delivery after 37 weeks of gestation.

Women were included if they were aged between 18 and 45 years old, with a singleton pregnancy, midpelvic cephalic presentation at the beginning of expulsive efforts and spontaneous occiput anterior delivery.

Women who refused to participate or were deprived of liberty by judicial or administrative decision or under legal protection were not included.

### Data collection

An electronic case report form was used to collect demographic and clinical data on the woman and the new born. Maternal and neonatal serious events were collected.

### Intervention

**Fig. 1:**
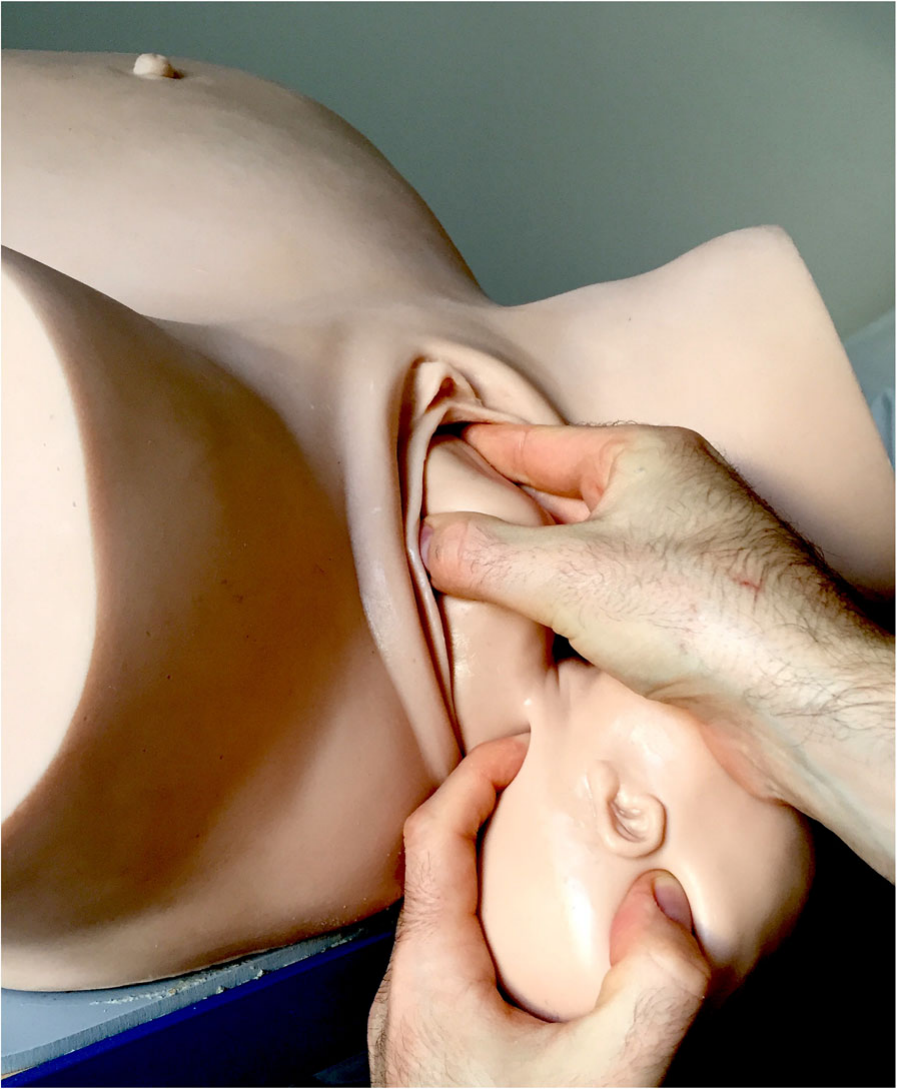
Index and middle fingers insertion with the hand opposite the fetal face [NM]

**Fig. 2:**
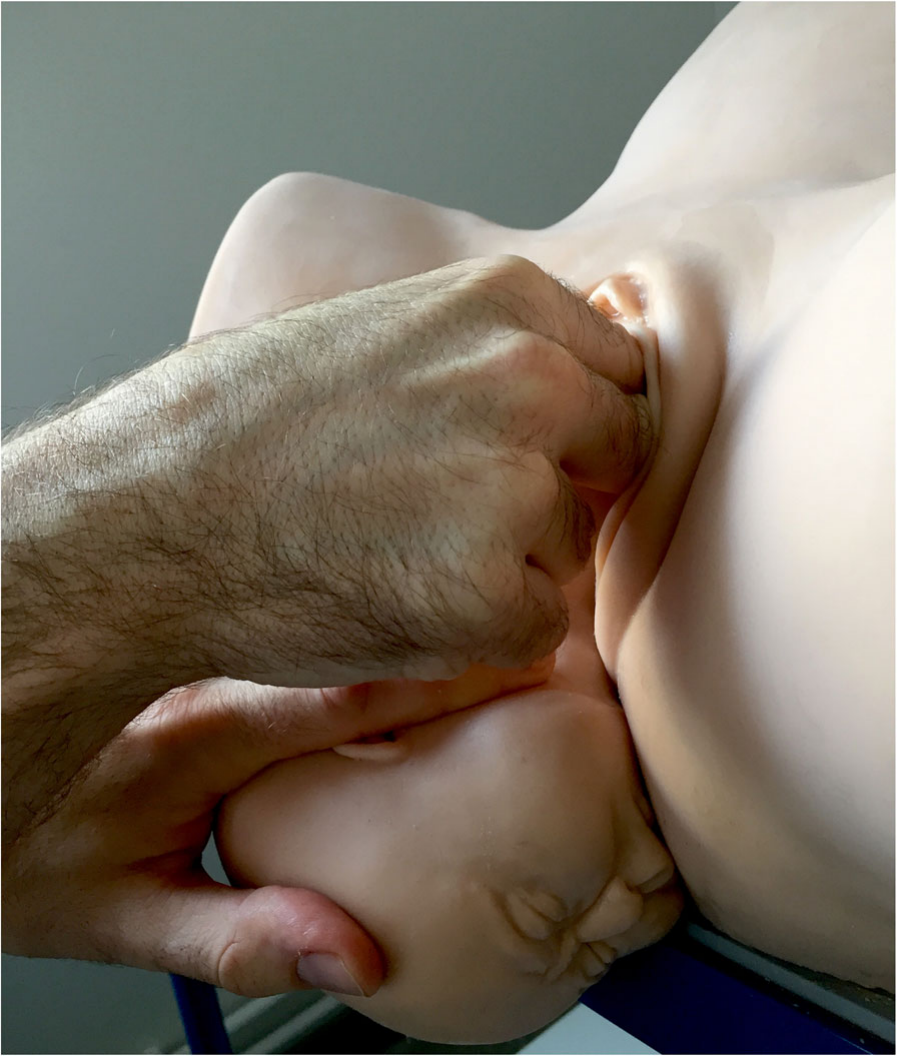
Fetal head slightly tilted downward with the free hand [NM]

**Fig. 3:**
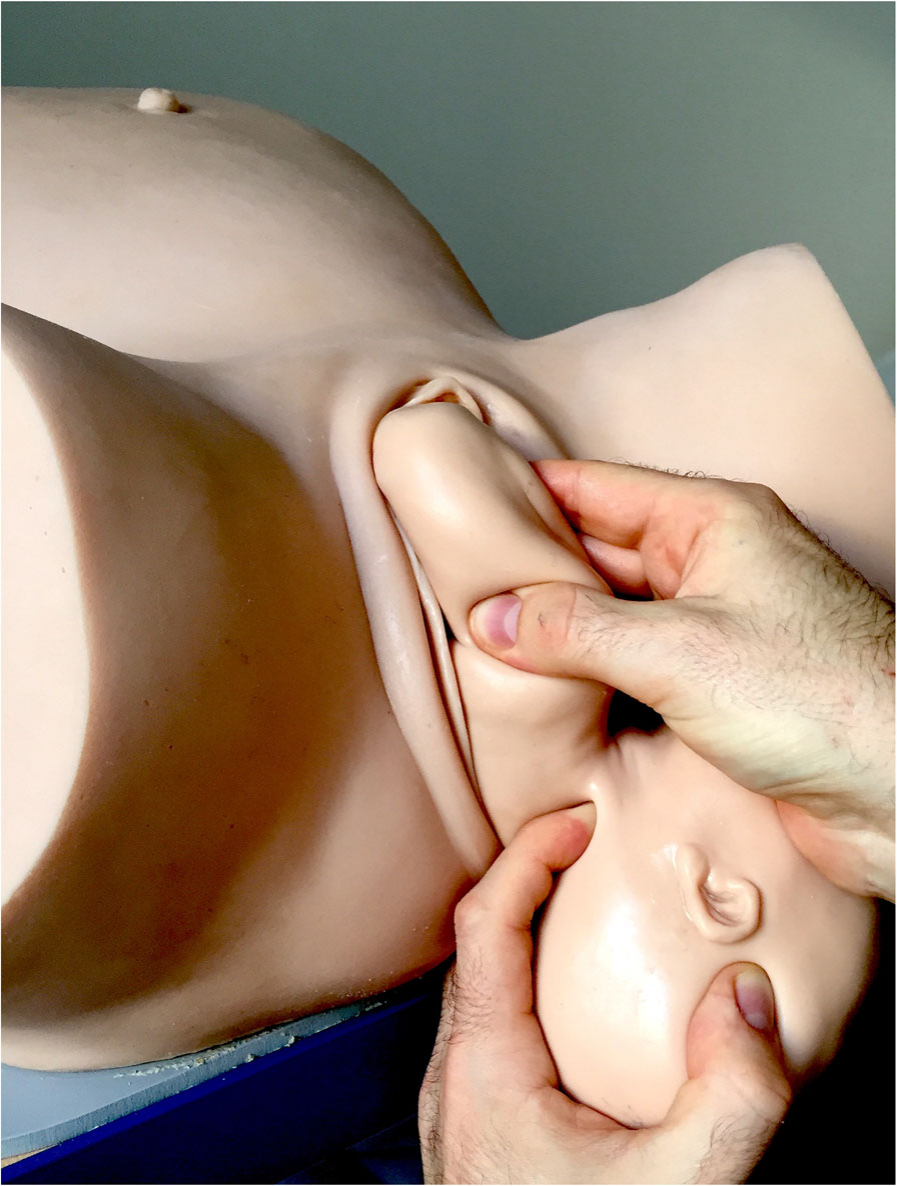
Two fingers are placed on the humerus like a splint [NM]

**Fig. 4:**
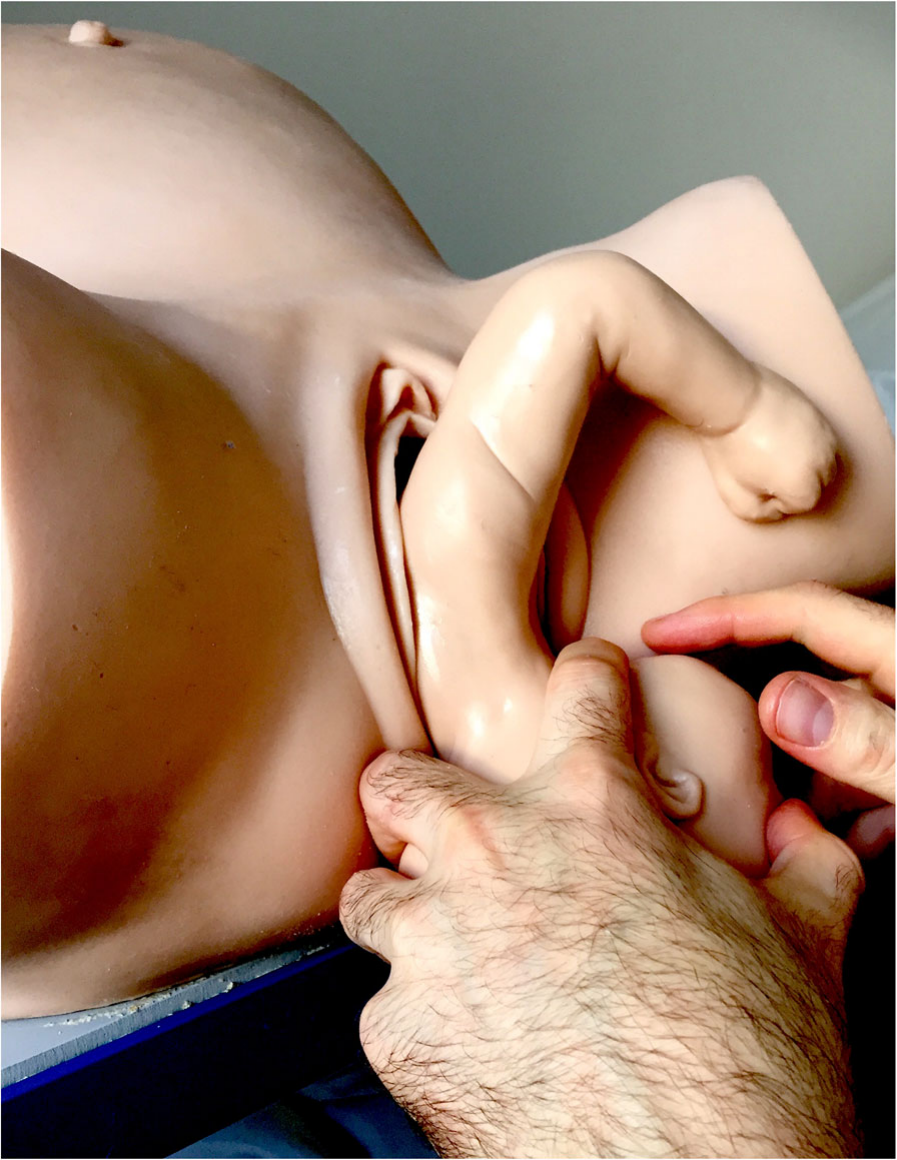
Fetal hand appears under the maternal pubic symphysis, allowing the anterior arm to be delivered [NM]

**Fig. 5:**
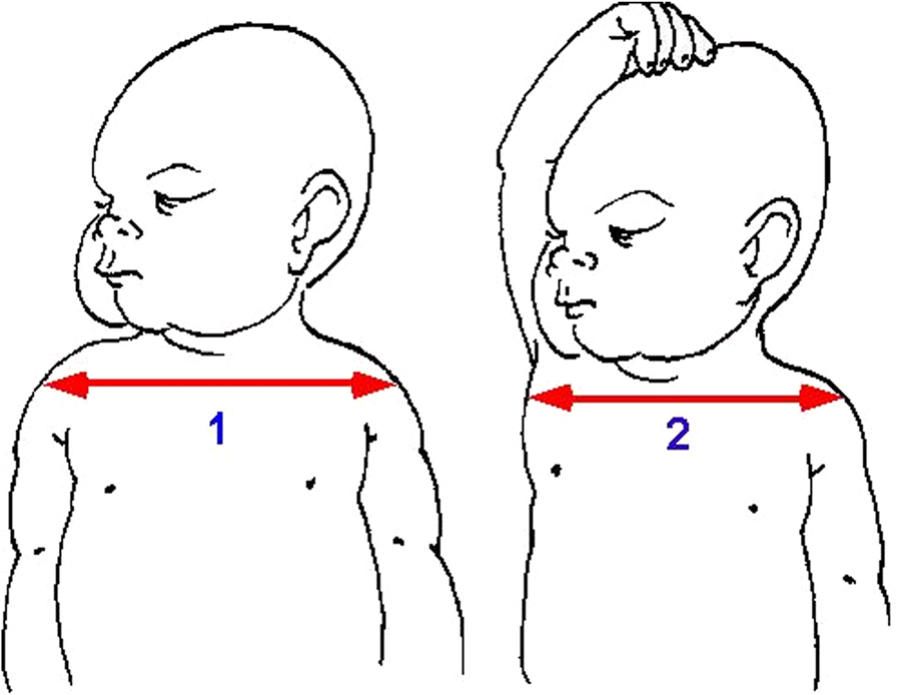
Reduction of bisacromial diameter into acromio-thoracic diameter, leading to a 30 mm decrease [NM]

In our center, CM is usually performed during normal deliveries. In CM, the anterior fetal arm is manually brought down as the shoulders are being delivered. The maneuver, which was initially described for treating shoulder dystocia, has the advantage of reducing fetal biacromial diameter. When there is insufficient room to deliver the anterior shoulder with the posterior shoulder in the pelvis, the anterior arm can be delivered, which brings the anterior shoulder under the pubis [15]. The birth attendant inserts the index and middle fingers of the hand opposite the fetal face under the mother’s pubic symphysis along the fetal humerus (Fig. 1). The fetal head is slightly tilted downward with the free hand (Fig. 2). The two fingers are placed on the humerus like a splint and the arm is pushed toward the fetal back (Fig. 3). The fetal hand then appears under the maternal pubic symphysis, allowing the anterior arm to be delivered (Fig. 4). The diameter pushing down on the posterior perineum is thus reduced from 13 cm (biacromial diameter) to less than 10 cm (axillo-acromial diameter) (Fig. 5). Midwives and obstetrician in our maternity are accustomed to practice this maneuver at delivery to protect perineum.

In this study, midwives performed CM and were asked to judge the difficulty of the maneuver: easy, moderately easy or difficult. They were trained to safely practice this technique. Two training sessions took place before the study. One recapped the principle behind the maneuver and the other involved training on a birthing simulator (MODEL-med “Sophie and her Mum Full Birth Obstetric Trainer”). All midwives were also trained in the evaluation and classification of perineal tears.

### Outcomes

The primary outcome was the success rate of the maneuver. Secondary outcomes were types of perineal tears according to the Royal College of Obstetricians and Gynaecologists’ classification [16], and neonatal birth trauma (fracture of the clavicle and humerus). Absence of fracture was checked by a pediatric examination during the maternity stay.

### Statistical analysis

**Fig. 6:**
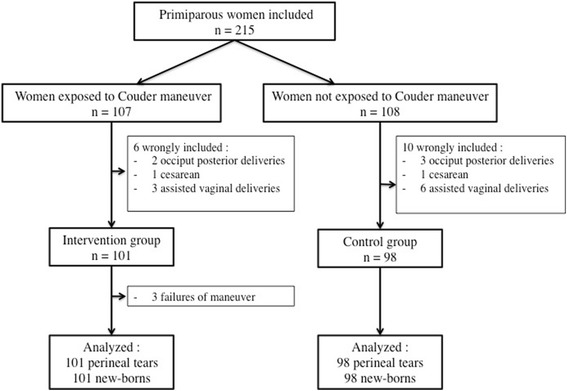
Study flowchart

To ensure that this study was statistically reliable, we estimated how many subjects were needed using the tool BioStat®. Prospective studies have shown that second degree occur in 13-36% of women who deliver vaginally [5, 17–22] . Based on our usual rates of perineal trauma in nulliparous women, we hypothesized that the frequency of second-degree tears would be 28% in the non-exposed group and 10% in the exposed group [23]. No studies in the literature are available on the rate of perineal tears after CM. Allowing for an alpha risk of 5% and a power of 90%, the number of subjects that was required in each group was at least 98. In some cases of emergency, some women would be included too early without respect of inclusion criteria (incorrect diagnosis of fetal head station or presentation for example). These situations were not protocol deviation but inclusion errors independent of the treatment or its outcome. Thus, the intention to treat analysis was performed for all “well randomised” women after exclusion of wrongly included women (Fig. 6).

Descriptive statistics were calculated, including frequencies and means. Continuous data were compared using a *t*-test if the variable was normally distributed or Mann Whitney test for non-parametric variables. The Chi-square test (Fisher’s exact test if necessary) was used for categorical variables. Statistical significance was considered at *p*-value ≤0.05. Data were analyzed with SAS 9.3 for Windows (SAS Institute Inc., Cary, NC, USA).

## Results

A total of 107 consecutives women exposed to CM were included and compared with 108 included in the control group. Sixteen women who in the end did not meet the inclusion criteria were excluded (9 assisted births before the beginning of expulsive efforts, 5 occiput-posterior delivery 2 cesareans during labor,). In these 9 cases of assisted delivery, a vacuum was used for non-reassuring fetal status before the beginning of expulsive efforts. Finally, two groups were compared: 101 consecutive women in the intervention group compared to 98 consecutive control women. The final number of patients was therefore 199.

As presented in the Table 1, clinical and obstetrical characteristics were not different between the two groups. The average bi-acromial diameter and birth weight of the children in each group were similar and birth weight values ranged from 2340 g to 4350 g. There was no difference in the distribution of birth weight and Apgar score in the two groups.

**Table 1:** Maternal and new-borns characteristics

	Couder group (*n* = 101)	Control group (*n* = 98)	*p*-value
Maternal characteristics:			NS
Maternal age (years)	26.9	27.8	
BMI (kg/m^2^)	21.5	22.1	
Weight gain (kg)	13.6	13.7	
Comorbidities:			
-Diabetes	7 (6.9%)	8 (8.1%)	
-Hypertension	2 (1.9%)	1 (1%)	
-Others	10 (9.9%)	8 (8.2%)	
New borns characteristics:			NS
Mean birth weight (g)	3228 [2880–4350]	3129 [2340–3910]	
Biacromial diameter (mm)	123 +/- 10	121+/− 9.5	
Apgar score <7 (5 min)	3 (3%)	2 (2%)	

*BMI* Body mass index

In the intervention group, 3 (3%) failures of the maneuver were reported. The maneuver was considered easy in 80% of cases, moderately easy in 12% and difficult in 8% of cases. Any clavicle or humerus fracture was recorded after a pediatric examination.

There was a significant difference concerning the secondary criteria. We found a significant difference (*p* = 0.03) in the rate of perineal tears between the two groups. No episiotomy was performed in this study. We found a significant reduction in the number of second-degree perineal trauma in the patients exposed to CM (*p* < 0.01) and a significant increase in first-degree tears (*p* = 0.03) (Table 2). No third and no fourth degree perineal tears were found. The number of intact perineums was higher in the group exposed to CM, but there was no significant difference. All the second-degree perineal tears avoided by CM were thus uniformly distributed in first-degree perineal tears or intact perineum. When there was second-degree trauma of the anal triangle, there was no difference in birth weight between the two groups (3380 g vs. 3370 g). We did not observe any significant difference (*p* = 0.33) in the rate of anterior perineal trauma between the exposed arm (43.6%) and non-exposed arm (36.7%) (Additional file 1).

**Table 2:** Impact of Couder’s maneuver on perineal lesion types

	Couder group (*n* = 101)	Control group (*n* = 98)	*p-*value
Posterior perineum:			
- Intact perineum	21 (20.8%)	14 (14.3%)	NS
- First degree	71 (70.3%)	55 (56.1%)	0.03
- Second degree	9 (8.9%)	29 (29.6%)	<0.001
Anterior perineum:	44 (43.6%)	36 (36.7%)	NS
- Labial tears	38 (86.4%)	32 (88.9%)	
- periurethral tears	6 (13.6%)	4 (11.1%)	

## Discussion

### Main findings

Couder maneuver in primiparous women at term is feasible with a low failure rate and influences the distribution of perineal tears by lowering second-degree perineal tear in a highly significant manner (p <0.01). In our series, there were three times less second-degree perineal lesions in the patients who were exposed to this maneuver. These avoided lesions became instead first-degree perineal trauma or intact perineum. Although our study did not show any significant differences in the rates of intact perineums between the two groups, our exposed group’s rate of 20.8% was much higher than the 9.6% reported in a 2013 study in nulliparas [5]. Again, suturing such slightly damaged perineum can be discussed in the interest of optimizing the perineal comfort of patients. Several studies show that not suturing the skin reduces perineal pain and dyspareunia, without increasing the risk of the wound’s reopening [23]. No third- or fourth-degree perineal tears were observed during this study, which is in line with the very low rate in our department – respectively, 4% and 2% based on our 2010 study [24]. There was no significant difference in the rate of anterior perineal lesions in the two groups (*p* = 0.33).

This maneuver does not aim to overmedicalize childbirth. It should be assimilated as a continuation of the maneuver of lowering the fetal head and aiding the natural movement of restitution. Indeed, in the lithotomy birthing position, and unlike the lateral or upright positions, in which the posterior shoulder is delivered first, it is the manual assistance of the movement of restitution, which, by tensing the sternocleidomastoid muscle, leads to the delivery of the anterior shoulder, first. CM is simply the logical continuation of managing this anterior upper limb by completely lowering it under the pubic symphysis. Lowering the anterior arm in a calm and controlled fashion does not in any way delay fetal expulsion. The fetal head must be delivered carefully to manage the perineum as to avoid severe perineal trauma, but carefully managing delivery of the shoulders also contributes to reducing lesions.

The failure rate was estimated at 3%. Even though no cases of obstetric fracture were reported in our series, this risk does exist, in particular for the humerus, and it results from poor execution and pulling perpendicularly to the axis of the arm. The prognostic for these fractures is, however, very good. Immobilization is most often all that is required, and remodeling is complete after 6 months [25, 26].

### Strengths and limitations

This study provides an objective view of our clinical practice. The design is simple with a significant statistical power demonstrating the feasibility and benefit of this maneuver. In a previous study, we reported that our policy of restricting the use of episiotomy did not increase the risk of third- and fourth-degree perineal tears, with a rate of episiotomy of barely more than 1% [6]. This policy of highly selective use led to a statistically significant increase in the rate of intact perineums, allowing more than 85% of patients who gave birth vaginally to leave our maternity department with trauma that was less severe than that associated with episiotomy. Thus, results of this present study are not applicable in all maternity wards where episiotomy is regularly performed.

Futhermore, the use of CM must be taught and supervised in a non-trained team. Indeed, operators who use it to early, based on a misunderstanding or anxiety can interfere with normal restitution and interfere with normal shoulder delivery. Training sessions are necessary to safely practice this technique in delivery rooms. In our study, professionals who took part to the study were volunteer and accustomed to practice this maneuver at delivery. External validity could be lost when CM is performed without prior teaching.

Others limitations of the study are absence of blinding and randomization. Lack of blinding can lead to the operator classifying tears as not in need of suturing. They may classify small second-degree tears as first degree. In our teaching maternity, three professionals are present with the parturient in the delivery room: a midwife, a student midwife and a child care assistant. It was difficult for ethics reasons to propose a perineal examination by a fourth blinded person while preserving women’s intimacy after delivery. Intimacy has to be ensured according to the French hospitalized patient Charter [27]. Perineal tears could have been photographed before and after CM, but the interpretation would have been difficult and not contributive. No randomization was performed for this study because it was a prospective collection of data following the introduction of a new maneuver in current practice. It was necessary to ensure the feasibility of the maneuver by midwives before considering a randomised trial.

### Comparison with published data

Different means of prevention have been reported in the literature. According to the Cochrane database, involving four studies with a total of 2480 patients, performing a prenatal perineal massage reduced the incidence of perineal tears requiring suturing, with RR = 0.91 (CI 95%: 0.86–0.96) [28]. This difference was only significant in patients who had never delivered vaginally. However, no difference in the incidence of first- and second-degree perineal tears was found. Ruckhaberle et al. have reported a significant increase in the rate of intact perineums (37.4% vs. 25.7%; *p* = 0.05) in patients using an intravaginal balloon called EPI-NO® from 37 weeks of gestation onward [29]. Using it daily progressively dilates the vagina, the aim being to ensure better perineal stretch during delivery. However, there was no significant difference in the reduction of first- and second-degree perineal trauma (*p* = 0.81) rates in that study. The results on the use of hyaluronic acid are contradictory. According to Scarabatto et al., in a randomized prospective study in 139 nulliparae, there was a significant reduction in perineal trauma (39.4% vs. 76.5%) when this product was used [30]. Conversely, Colacioppo et al. found that its use did not reduce the rate of perineal tears and did not increase the number of intact perineums in a randomized prospective study [31]. Given our results, CM could be performed in nulliparae who do not undergo episiotomy for delivery of the fetal head in order to reduce the rate of second-degree perineal tears.

The extent of muscle tear is correlated with the extent to which the muscle fibers are stretched [32-34]. In CM, the posterior shoulder exerts less pressure on these muscles, which is therefore a further advantage. The degree of morbidity is directly linked to the degree of perineal damage, and damage to the puborectal fascicles influences urinary and anorectal function [13]. For Rogers et al., patients who suffer perineal muscular trauma during childbirth require more perineal rehabilitation sessions (OR = 3.06; CI 95%: 1.41–6.63) [35]. The integrity of the muscles and ligaments that make up the pelvic floor is crucial for ensuring sufficient pelvic floor equilibrium after childbirth [36]. It is recognized that any trauma of the levator ani muscle increases the risk of pelvic organ prolapse [37]. Muscle tear is often accompanied by nerve trauma that may worsen both disorders of the pelvic floor and urinary sphincter functions [38, 39]. According to a case–control study in 68 patients with anal incontinence, Bharucha et al. revealed that puborectal muscle trauma was significantly associated with a deterioration of voluntary sphincter contractility (*p* = 0.05) [40]. All these dysfunctions significantly lower patient’s quality of life [41]. These results show why it is essential to avoid second-degree perineal trauma during childbirth, especially in nulliparae, who are recognized as being at risk of perineal trauma [42].

## Conclusion

This is the first study reporting a means of significantly reducing the incidence of second-degree perineal lesions without recourse to episiotomy, and it is the first series describing the use of CM in nulliparous patients. The maneuver improves the overall rate of perineal tears, reducing second-degree perineal tears in a highly significant manner (*p* < 0.01) without increasing trauma to the urogenital triangle. It thus lowers the number of sutures required. This study may provide a rationale for systematically using CM in nulliparous patients when an episiotomy has not been performed. These results encourage continuing and improving this work with a future randomized study.

## Additional file

Additional file 1: Descriptive statisitics of the study. (XLS 76 kb)

## Abbreviations

CM: Couder’s Maneuver
BMI: Body mass index
